# Retrograde Gastrojejunostomy Tube Migration

**DOI:** 10.1155/2014/738506

**Published:** 2014-12-29

**Authors:** Adeleke Adesina, Guhan Rammohan, Rebecca Jeanmonod

**Affiliations:** St. Luke's University Hospital, 801 Ostrum Street, Bethlehem, PA 18015, USA

## Abstract

Percutaneous enteral feeding tubes are placed about 250,000 times each year in the United States. Although they are relatively safe, their placement may be complicated by perforation, infection, bleeding, vomiting, dislodgment, and obstruction. There have been numerous reports of antegrade migration of gastrojejunostomy (G-J) tubes. We report a case of G-J tube regurgitation following protracted vomiting and discuss the management of this very rare entity.

## 1. Case Presentation

A 33-year-old male presented to the emergency department (ED) for severe vomiting for the past several hours. The patient stated that the vomiting was sudden in onset and forceful and episodic and nonbloody and nonbilious in nature. The patient had a history of gastroparesis secondary to poorly controlled diabetes and had episodic forceful vomiting leading him to present to the ED on numerous prior occasions. On this occasion, after several vomiting episodes, the patient noted the tip of his MIC gastrojejunostomy (G-J) tube protruding out his mouth. The patient also complained of chest and epigastric pain. He denied fever, shortness of breath, or diarrhea. The G-J tube had been placed 3 months ago for severe diabetic gastroparesis. The patient had had no complications from the procedure and he had been successfully giving himself feeds through his jejunal port and draining his gastric port. The patient's past medical history also included poorly controlled insulin-dependent diabetes, end stage renal failure, and prior thromboembolic disease.

**Figure 1:**
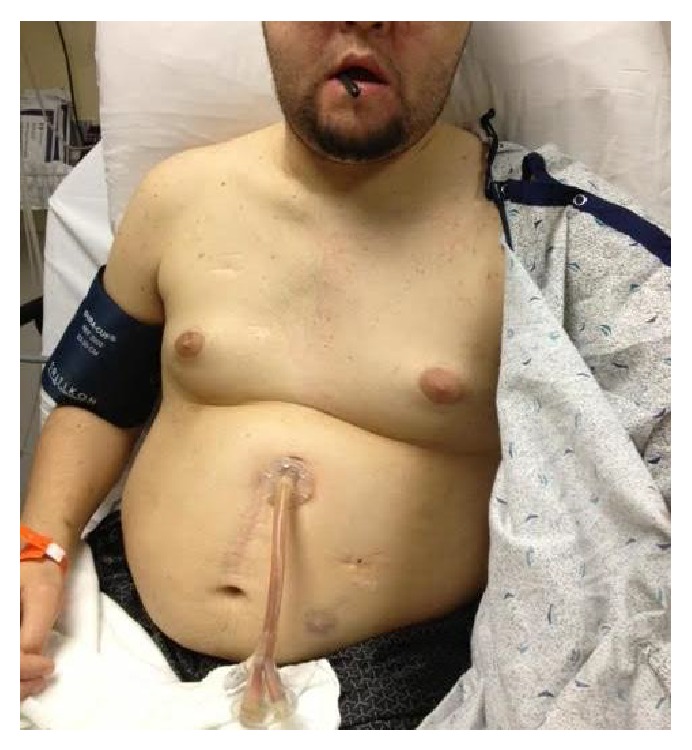
Patient with the tip of the G-J tube protruding from his mouth.

On examination, the patient appeared to be in moderate distress, gagging on the G-J tube with the tip extruding out of the mouth (Figure 1). He otherwise had a normal head and neck exam, with no evidence of subcutaneous air or tracheal deviation. His heart was regular without rub, and his lungs were clear bilaterally. His abdomen was soft with mild epigastric tenderness. He had no rebound or guarding. The remainder of his physical examination was unremarkable.

**Figure 2:**
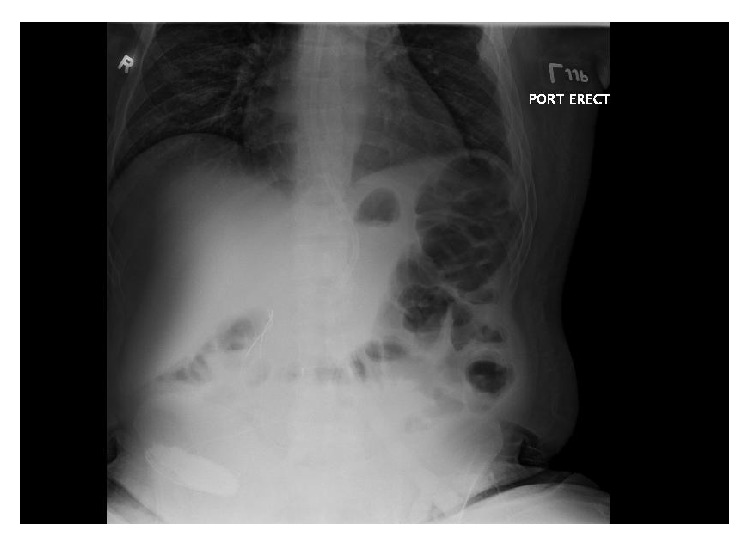
Portable chest radiograph demonstrating jejunostomy tube extending retrograde through esophagus, with no evidence of perforation.

**Figure 3:**
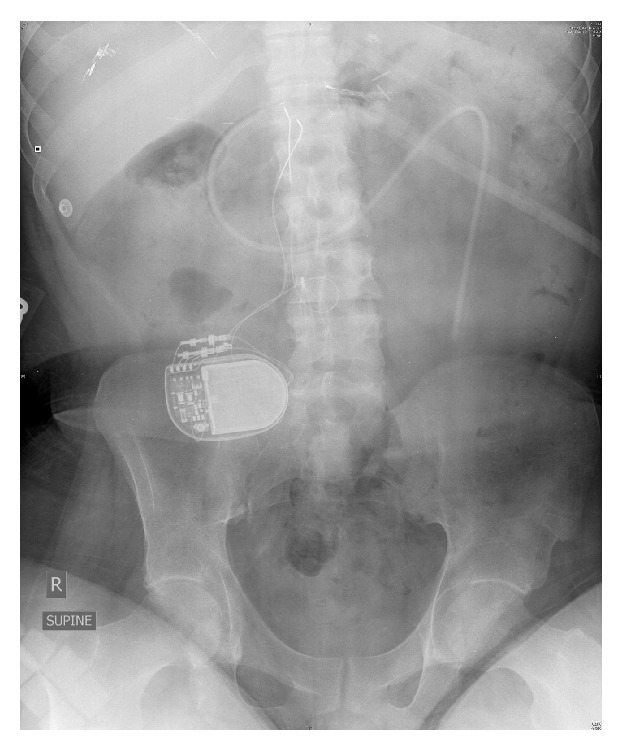
Supine radiograph demonstrating G-J tube in normal position.

A portable chest radiograph was performed to assess the position of the G-J tube as well as to look for evidence of gastrointestinal tract perforation. It demonstrated retrograde position of the tube from the stomach to the mouth with no mediastinal air or pneumothorax (Figure 2). The patient was given antiemetic medications and anxiolytics and the G-J tube was pulled back slowly from the percutaneous skin site and reduced into the stomach. This resulted in immediate relief of the patient's symptoms. He subsequently underwent G-J tube repositioning under fluoroscopic guidance by interventional radiology (Figure 3).

## 2. Discussion

Percutaneous placement of enteral feeding tubes was first performed in 1979 and has become the placement method of choice for patients requiring feeding tubes [1, 2]. Although gastric tubes are more commonly placed, these may result in aspiration, particularly in patients with abnormal gastric anatomy or function [2]. In these patients, jejunostomy or gastrojejunostomy (in which one port is used to feed through the jejunum and the other is used to drain the stomach) is preferred [2, 3].

Like the placement of percutaneous gastric tubes, G-J tube placement may be associated with major complications, such as sepsis, hemorrhage, buried bumper, and gastrointestinal perforation, or minor complications, such as tube dislodgement, leaking, diarrhea, and vomiting [4–6]. In addition to these, gastrojejunostomy tubes have more commonly been associated with obstruction, intussusception, and antegrade enteral tube migration [7–9]. Antegrade migration is uncommon, as typically there is suture or a bolster holding the tube at the skin entry site. However, should the suture erode through the skin or the bolster become dislodged, bowel peristalsis may propel the tube into the GI tract before the patient or caregiver realizes its absence. These cases are typically managed expectantly unless bowel obstruction occurs.

Retrograde migration out the mouth, which has not been previously reported, is more difficult to explain. One possible mechanism is that, with forceful vomiting or other conditions that increase intra-abdominal pressure, the pressure gradient between the abdomen and the thorax encourages the tube to migrate in a retrograde fashion. Since most patients with G-J tubes have gastric dysmotility and reflux, the tube is unlikely to spontaneously return to its desired position in the jejunum once it has dislodged to the stomach. With forceful vomiting, as with our patient, the tube was likely simply refluxed into the esophagus and extruded through the mouth.

Prevention of G-J tube migration is best accomplished by proper G-J tube positioning with adequate tube length extending into the jejunum [10]. It is important to not leave any redundant tubing in the stomach, as any loop of tubing increases the risk of tube migration into the stomach [10]. Instead, the tube should extend directly through the pylorus with no laxity.

Initial management of this entity should begin with the assessment of the patient's airway. Although there is currently no evidence that orogastric intubation increases aspiration risk [11], large studies have not been performed. Certainly, a foreign object in the posterior oropharynx causes gagging and nausea, which may lead to vomiting and aspiration.

Once the airway has been assessed, it is important to define the location of the tube with physical exam, radiographically. Although perforation of the gastrointestinal tract is a known complication of initial placement of feeding tubes, perforation secondary to migration of gastrojejunostomy tube is rare but has been described [12]. Therefore, it is necessary for the provider caring for the patient to assess for this possibility when evaluating a tube which has migrated. Furthermore, since perforation of the gastrointestinal tract has been reported as a complication of jejunal tube removal [13], it is wise to assess for perforation prior to removal. A patient with no peritoneal findings on abdominal exam and normal lung exam with a normal upright chest or decubitus film is likely sufficient, although CT scanning can be used in unclear cases.

Patients with uncomplicated G-J tube migration should, when possible, have their tubes redirected or replaced with fluoroscopic guidance. If this is not available, reduction of the tube into the stomach or complete removal of the tube should be undertaken. To prevent tube tract stenosis, a foley catheter should be placed into the tract temporarily. Patients in whom a G-J tube has been reduced into the stomach should not undergo tube feeding, as they are at high risk for aspiration. Instead, the tube should be placed to drainage and they should be given supplemental intravenous fluids for maintenance of hydration and admitted pending tube replacement. Patients with G-J tube migration complicated by perforation should receive broad spectrum of antibiotics, intravenous isotonic fluid resuscitation for volume depletion, and immediate surgical evaluation.

## 3. Conclusion

Patients with percutaneous gastrostomy and G-J tubes are routinely encountered in the emergency medicine practice. In addition to the more common complications, the emergency provider should be familiar with feeding tube migration and its management.
